# A Novel Autonomous 5-D Hyperjerk RC Circuit with Hyperbolic Sine Function

**DOI:** 10.1155/2018/1260325

**Published:** 2018-09-03

**Authors:** N. Tsafack, J. Kengne

**Affiliations:** ^1^Research Unit of Laboratory of Automation and Applied Computer (LAIA), Electrical Engineering Department of IUT-FV, University of Dschang, P.O. Box 134, Bandjoun, Cameroon; ^2^Research Unit of Laboratory of Condensed Matter, Electronics and Signal Processing (URMACETS) Department of Physics, Faculty of Sciences, University of Dschang, P.O. Box 67, Dschang, Cameroon

## Abstract

A novel autonomous 5-D hyperjerk RC circuit with hyperbolic sine function is proposed in this paper. Compared to some existing 5-D systems like the 5-D Sprott B system, the 5-D Lorentz, and the Lorentz-like systems, the new system is the simplest 5-D system with complex dynamics reported to date. Its simplicity mainly relies on its nonlinear part which is synthetized using only two semiconductor diodes. The system displays only one equilibrium point and can exhibit both periodic and chaotic dynamical behavior. The complex dynamics of the system is investigated by means of bifurcation analysis. In particular, the striking phenomenon of multistability is revealed showing up to seven coexisting attractors in phase space depending solely on the system's initial state. To the best of author's knowledge, this rich dynamics has not yet been revealed in any 5-D dynamical system in general or particularly in any hyperjerk system. Pspice circuit simulations are performed to verify theoretical/numerical analysis.

## 1. Introduction

The study of three-dimensional dynamical systems seems to be mature [1–15]. The interest is now focused on high dimensional systems [16–21]. The reason is that they have been discovered to model natural phenomena more explicitly than the three-dimensional dynamical systems. Recently, hyperjerk systems have received great attention. In 2005, Konstantinos and Sprott proposed a class of chaotic/hyperchaotic hyperjerk systems [19]. They investigated the dynamical properties of the proposed systems focusing on potential chaotic and hyperchaotic dynamics. They also claimed to have proposed surprisingly simple and most elegant functional forms of hyperjerk systems. A year later, Stefan showed that the concept of hyperjerk systems might serve as an appropriate starting point to study the dynamics of driven or coupled oscillators in a unified way [22]. The five-dimensional (5-D) hyperchaotic Sprott B system has ten terms including two quadratic nonlinear terms and only one control parameter. Hong Mey and collaborators have recently proposed a novel cryptosystem based on 5-D hyperchaotic system [23]. The system is combined with the logistic map for the generation of pseudo random sequences of better properties. In order to analyze the behavior of chaotic systems, some researchers focused on the synchronization and control of the 5-D systems [23–26]. These systems are algebraically simpler than Lorenz and Lorenz-like 5-D systems with twelve terms, three quadratic nonlinear terms including five or six control parameters. Let us note that the practical realization of quadratic nonlinearity found in these works is expensive due to the presence of analogue multipliers. Recently multistability has attracted tremendous research efforts [1, 5, 12]. However to the best of the author's knowledge, multistability in 5-D hyperjerk systems is very little documented. Also it is very interesting to question whether there exists a simple chaotic 5-D hyperjerk system capable of multiple coexisting attractors.

This paper investigates the dynamics of a novel 5-D hyperjerk circuit with a very simple nonlinear part (a pair of semiconductor diodes). The new circuit can be regarded as a 5-D version of the jerk circuit previously reported by Kengne and collaborators [27]. It is important to stress that the simplicity of the 5-D autonomous circuit relies on the practical standpoint where the nonlinear part is constructed using semiconductor diodes. Despite the relative simplicity of their electronic circuits, the proposed circuit is characterized by rich and striking nonlinear behaviors such as chaos, antimonotonicity, multiple coexisting attractors.

The paper is organized as follows: Section 2 deals with the modeling process. The electronic structure of the oscillator is described and a suitable mathematical model is derived to investigate the dynamics of the system.  Section 3 is concerned with the numerical analysis. Various bifurcation diagrams combined with their corresponding graphs of the largest Lyapunov exponent are plotted to reveal different transitions to chaos. The occurrence of multiple attractors is also discussed using bifurcation diagrams as arguments. In addition the bubbling phenomenon is presented. Pspice circuit simulations results are carried out in Section 4. Some concluding remarks are presented in Section 5.

## 2. Presentation of the Novel 5-D Circuit

**Figure 1:**
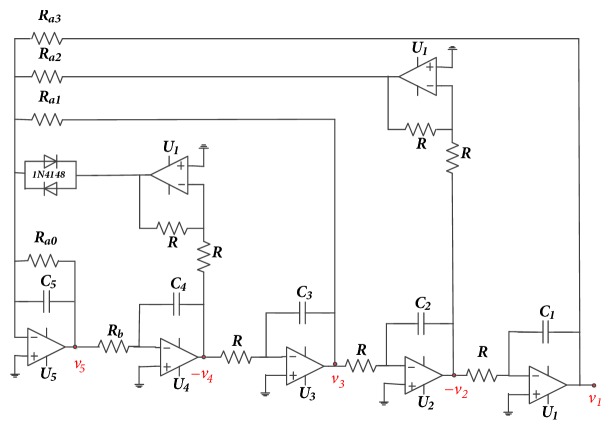
Electronic circuit of the proposed 5-D hyperjerk system.

The electronic circuit of the oscillator under investigation is depicted in Figure 1. It consists of five successive active integrators in a feedback loop. Additionally, another feedback loop involving two of the integrators and a pair of diodes (e.g., type 1N4148) connected in antiparallel is used to synthetize the nonlinear part. The pair of semiconductor diodes is the only element responsible for the complex dynamics of the circuit. For instance, up to seven disconnected attractors have been found depending solely on the system's initial state. Upon applying Kirchhoff's electrical circuit laws and the Shockley diode equation [26] to the circuit of Figure 1, the following set of five coupled first-order differential equations can be derived:

(1) C1dυ1dt=υ2RC2dυ2dt=υ3RC3dυ3dt=υ4RC4dυ4dt=υ5RbC5dυ4dt=−υ5Ra0−υ3Ra1−υ2Ra2−υ1Ra3−2Issinh⁡υ4ηVT

To ease the numerical analysis of the circuit, the following change of variable and parameters is considered:

(2) υi=xiVrefi=1⋯5;t=RCτ;Vref=ηVT;b=RRb;aj=RRajj=0⋯3;γ=2RISVref

Thus the dimensionless circuit equations are derived as follows:

(3) x˙1=x2x˙2=x3x˙3=x4x˙4=bx5x˙5=−a0x5−a1x3−a2x2−a3x1−γsinh⁡x4

The simplicity of the new 5-D autonomous system (3) studied in this paper mainly relies on its nonlinear part which is synthetized using only two semiconductor diodes. With reference to some chaotic systems in the literature [16–21], cubic or quadratic nonlinear product terms are mostly used. Accordingly, the practical construction is more complicated due to the use of multipliers. In addition, the maximum Lyapunov exponent of the studied 5-D chaotic system is bigger than that of some dynamical systems. Hence, it has more complex dynamic behaviors.  Table 2 is provided for more illustration.

System (3) is dissipative since its divergence (∇*V* = −*a*_0_) is negative. Consequently, all system orbits will be confined to a specific bounded subset of zero volume in state space and the asymptotic dynamics converges to an attractor. This is true for this particular case. However, the system is dissipative in the sense of Levinson if there exists nonlocal Lyapunov function which defines the absorbing set [23]. In addition, System (3) is invariant under the transformation: (x_1_, x_2_, x_3_, x_4_, x_5_)⇔(−x_1_, −x_2_, −x_3_, −x_4_, −x_5_). Consequently, if (x_1_, x_2_, x_3_, x_4_, x_5_) is a solution of system (3) for a given set of parameters, then (−x_1_, −x_2_, −x_3_, −x_4_, −x_5_) is also a solution for the same parameter set. This property is responsible for the variety of coexisting attractors in the system. Finally, it is found that the novel oscillator under investigations has only one equilibrium point that is the origin *E*_0_(0,0, 0,0).

## 3. System's Dynamics

### 3.1. Computational Method

To produce phase portraits, bifurcation diagrams, and Lyapunov spectrum, the system was solved using the classical fourth-order Runge-Kutta algorithm with the time step always Δ*t* ≤ 0.005. Two different methods are used to plot the various bifurcation diagrams in order to highlight the sensitivity of the whole system to the tiny changes of its parameters. The first method is by forward and backward continuations, i.e., using a properly continuation package for continuous-time systems [28], which is a standard tool for numerical bifurcation analysis. The second method is by following a specific attractor to obtain its behavior at long term. The later method is used to justify the abundant coexisting attractors in the system (up to seven coexisting attractors).

### 3.2. Route to Chaos and Antimonotonicity

**Figure 2:**
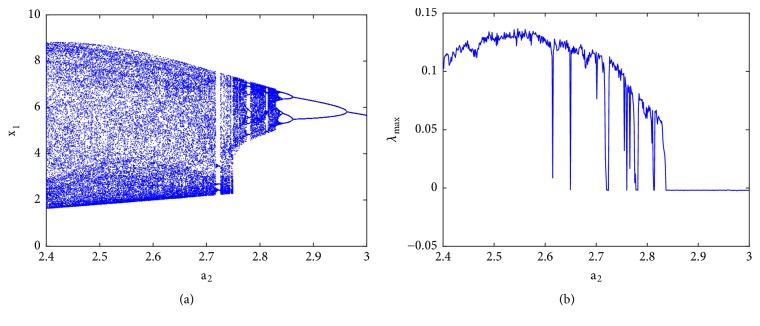
Backward continuation of system (3) when decreasing ***a***_2_ from 3 to 2.4 (a) and the corresponding graph of largest Lyapunov exponent (*λ*_max_) plotted in the range 2.4 ≤ ***a***_2_ ≤ 3 (b).

**Figure 3:**
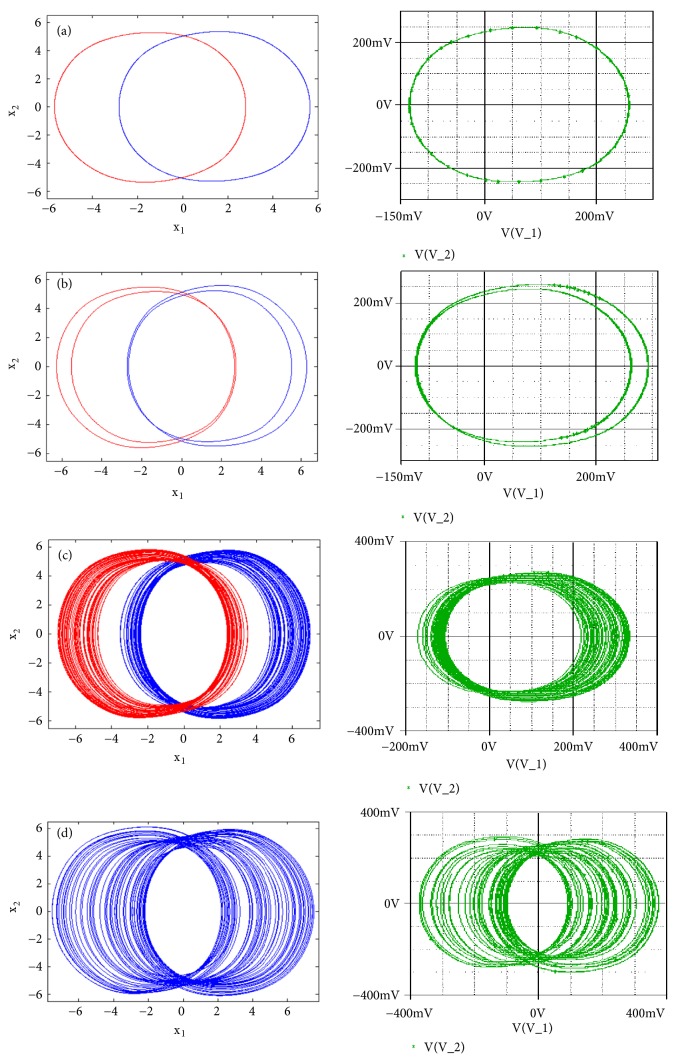
Numerical phase space trajectories (left) and Pspice based simulation results (right) showing the classical period doubling routes to chaos in the novel 5-D system.

To reveal the type of transition leading to chaos, a single control parameter (b) was considered to vary in the range 2.4 < *a*_2_ < 3 while the rest of system's parameters are fixed as follows: a_0_ = 1.75; a_3_ = 1; a_1_ = *b* = 3; *γ* = 0.0011. The bifurcation diagram of Figure 2 is obtained by forward continuation. From this diagram it is obvious that the system experiences the classical reverse period doubling bifurcation. It moves from period-1 limit cycle to double band chaotic attractors going through single band chaotic attractors and tiny windows of periodic attractors.  Figure 3 (left) illustrates this bifurcation sequence with the numerical phase portraits.

**Figure 4:**
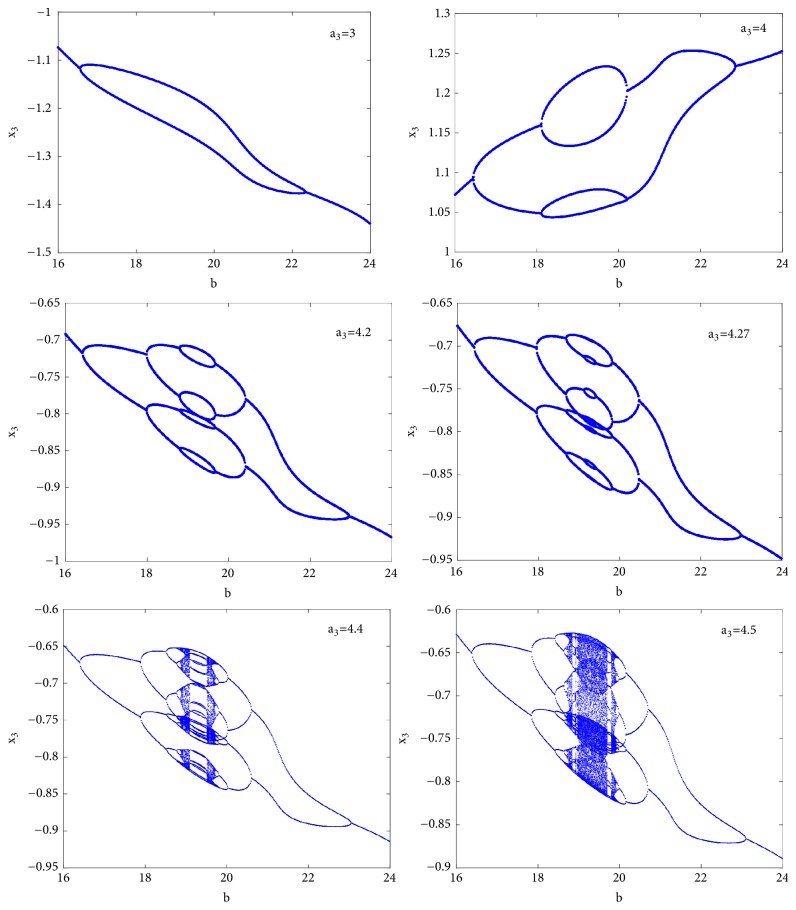
Bifurcation diagrams showing local maxima of the coordinate *x*_3_ of the attractor in Poincaré cross section in terms of the control parameter*** a***_2_ for remerging Feigenbaum tree (bubbling): period-2 bubble for a_3_ = 3, period-4 bubble for a_3_ = 4, period-8 bubble for a_3_ = 4.2, and full Feigenbaum remerging tree at a_3_ = 4.5.

As the system experiences the classical period doubling route to chaos, it is obvious that antimonotonicity can be observed. Represented on Figure 4, this behavior has been searched by varying parameter*** b*** in the range 16 to 24 for some discrete values of parameter a_3_. For instance, if a_3_ = 3 period-2 bubble is observed and each branch develops two branches leading to a stable period-4 bubble for a_3_ = 4. Similarly a period-8 bubble is obtained for a_3_ = 4.2. As the discrete parameter***b*** increases, more bubbles are created until an infinitely tree (chaos) finally occurs when a_3_ = 4.5.

### 3.3. Multiple Coexisting Attractors

Multistability has been previously revealed in many dynamical systems [29–34], including jerk systems with self-excited attractors [30, 33, 34] and hidden attractors [3, 6, 35]. But the models presented up to date achieved at most six coexisting attractors in the phase space. This behavior has not yet been discovered in any 5-D hyperjerk systems. The system under study can challenge this limit by displaying up to seven coexisting attractors depending solely on the system's initial state.

**Figure 5:**
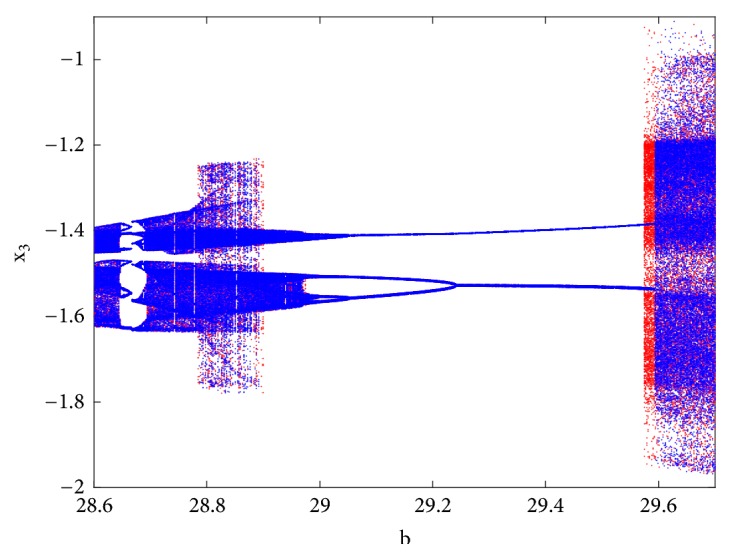
Bifurcation diagram for illustrating the coexistence of disconnected chaotic attractors with a pair of period-2 limit cycle. The diagram is plotted by forward or backward continuation of parameter b with the following initial conditions x_1_(0) = 3; x_2_(0) = x_3_(0) = x_4_(0) = x_5_(0) = 0 and x_1_(0) = 1; x_2_(0) = x_3_(0) = x_4_(0) = x_5_(0) = 0.

**Figure 6:**
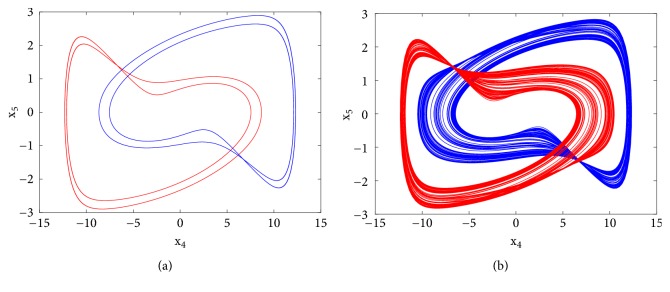
Two-dimensional projections (x_4_-x_5_) of four coexisting chaotic and periodic attractors for b = 29.57, a_0_ = a_2_ = a_3_, a_1_ = 7, a_4_ = 5.4433 x 10^−4^. Initial conditions are indicated in Table 1.

With reference to the bifurcation diagram of Figure 5 the classical forward or backward continuation of parameter b is obtained with the following initial conditions x_1_(0) = 3; x_2_(0) = x_3_(0) = x_4_(0) = x_5_(0) = 0 and x_1_(0) = 1; x_2_(0) = x_3_(0) = x_4_(0) = x_5_(0) = 0. A hysteretic window can be identified leading to the coexistence of four disconnected chaotic and periodic attractors (Figure 6) in the phase space for *b* = 29.57, *a*_0_ = *a*_2_ = *a*_3_ = 5, *a*_1_ = 7, *γ* = 5.4433*x*10^−4^.

**Figure 7:**
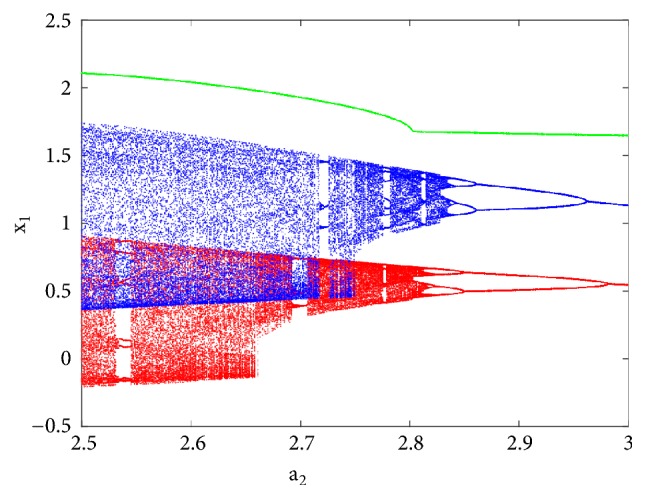
Bifurcation diagram for justifying the coexistence of four, five, and six different attractors in the phase space. The diagram in blue and red is plotted by forward and backward continuation while the diagram in green is plotted by following the attractor defined at a_2_ = 2.71 for x_1_(0) = 3; x_2_(0) = x_3_(0) = x_4_(0) = x_5_(0) = 0.

**Figure 8:**
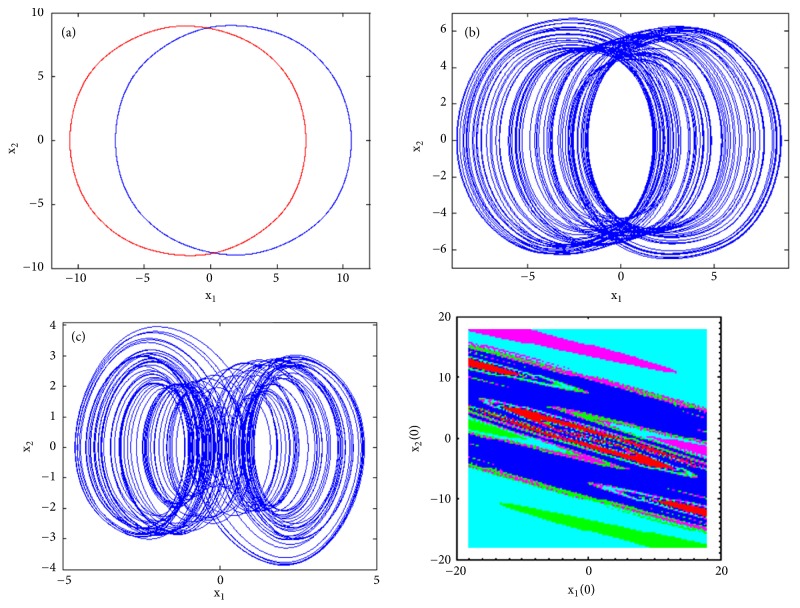
Coexistence of four different attractors (a pair of period-1 limit cycles and two different symmetric attractors) for a_2_ = 2.458 with the rest of system's parameters as follows: b = a_1_ = 3, a_0_ = 1.75, a_3_ = 1, a_4_ = 0.0054. Initial states are given in Table 1.

To observe more than four different attractors in the system, the second method described above (see Section 3.2) is used to plot the bifurcation diagrams of Figures 7 and 12 by varying parameter a_2_. If we fix systems parameters as a_2_ = 2.458; a_0_ = 1.75; a_1_ = b = 3; a_3_ = 1; *γ* = 0.0054, the system experiences four different periodic and chaotic attractors (see Figure 8) where the cross section of the basins of attraction of the attractors is also presented. This dynamics is justified using the bifurcation diagrams of Figure 7.

**Figure 9:**
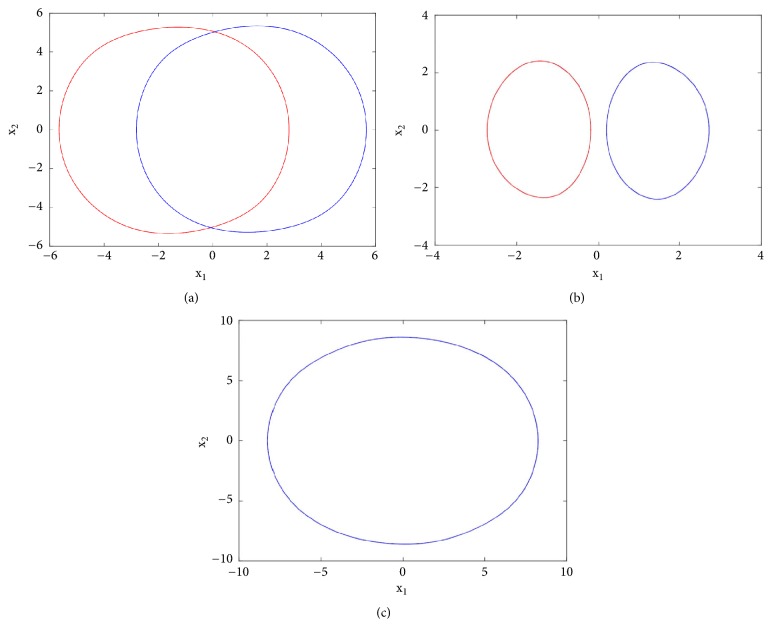
Coexistence of five different period-1 limit cycles for a_2_ = 3 with the rest of system's parameters as follows: b = a_1_ = 3, a_0_ = 1.75, a_3_ =1, a_4_ = 0.0054. Initial states are given in Table 1.

If systems parameters are a_2_ = 3; a_0_ = 1.75; a_1_ = b = 3; a_3_ = 1; *γ* = 0.0054, the system displays five different period-1 limit cycles (see Figure 9). This dynamics is justified using the bifurcation diagrams of Figure 7.

**Figure 10:**
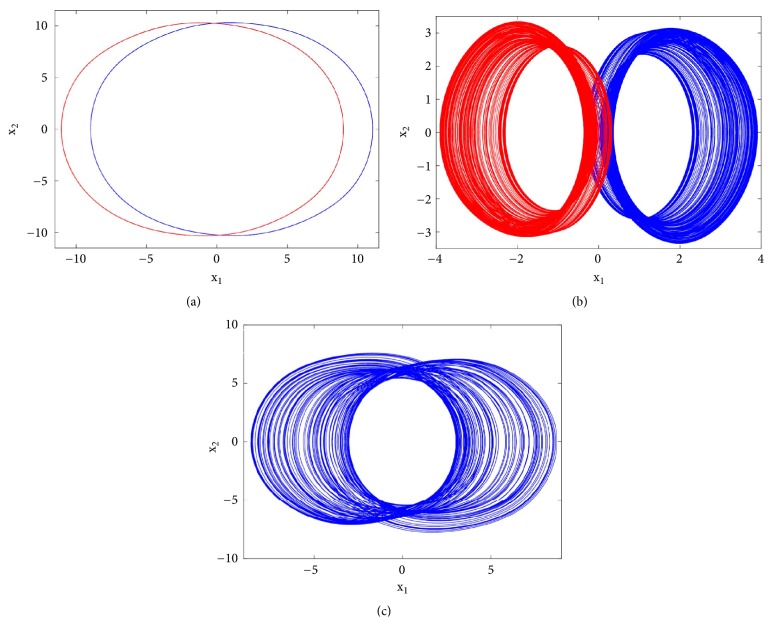
Two-dimensional projections (x_1_-x_2_) of five coexisting attractors for a_2_ = 2.71 (a pair of chaotic attractors, a pair of period-1 limit cycle, and a symmetric chaotic attractor) with the rest of system's parameters as follows: b = a_1_ = 3, a_0_ = 1.75, a_3_ = 1, a_4_ = 0.0054. Initial states are given in Table 1.

For a_2_ = 2.71; a_0_ = 1.75; a_1_ = b = 3; a_3_ = 1; *γ* = 0.0054, the system experiences five different periodic and chaotic attractors (see Figure 10). This dynamics is justified using the bifurcation diagrams of Figure 7.

**Figure 11:**
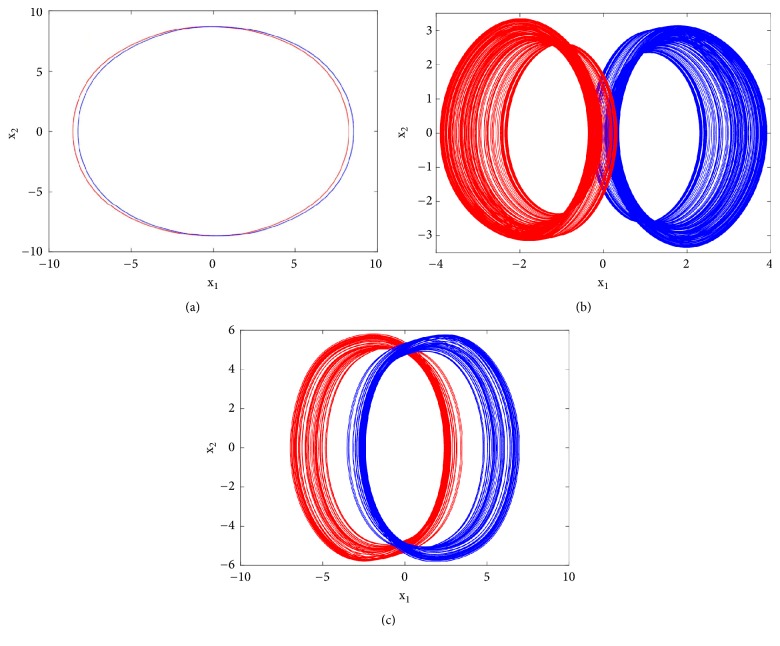
Two-dimensional projections (x_1_-x_2_) of six coexisting attractors for a_2_ = 2.8 (two pairs of chaotic attractors and a pair of period-1 limit cycle) with the rest of system's parameters as follows: b = a_1_ = 3, a_0_ = 1.75, a_3_ = 1, a_4_ = 0.0054. Initial states are given in Table 1.

For a_2_ = 2.8; a_0_ = 1.75; a_1_ = b = 3; a_3_ = 1; *γ* = 0.0054, the system displays six different periodic and chaotic attractors (see Figure 11). This dynamics is justified using the bifurcation diagrams of Figure 7. The corresponding attraction basin is shown in Figure 7. Let us note that an attractor is hidden if their basin of attraction does not intersect with small neighborhoods of the equilibrium points of system. Given that the single equilibrium point of system (3) *E*_0_(0,0, 0,0) intersects with the basin of attraction of the coexisting attractor Figure 7, we can conclude that the said attractor is self-excited (instead of hidden attractors). Some literature can provide systems with hidden attractors [3, 6, 35].

**Figure 12:**
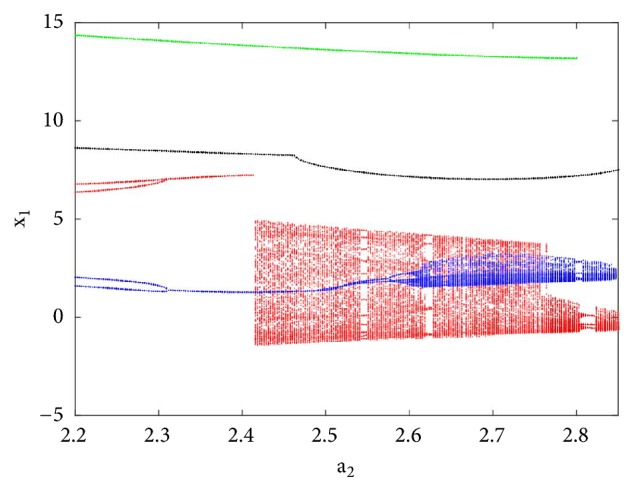
Bifurcation diagram for illustrating the coexistence of seven different attractors in the phase space. The diagrams are plotted using the same methods as in Figure 7.

**Figure 13:**
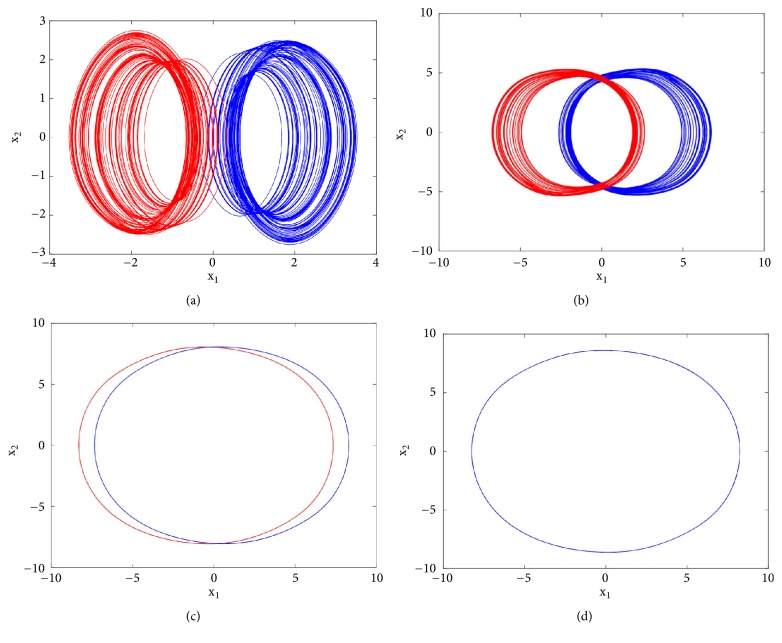
Coexistence of seven disconnected attractors (two pairs of chaotic attractors, a pair of period-1 limit cycle, and a symmetric period-1 limit cycle) for a_2_ = 3 with the rest of system's parameters as follows: b = a_1_ = 3, a_0_ = 1.75, a_3_ = 1, a_4_ = 0.0109. Initial states are given in Table 1.

More interestingly, another window of multiple coexisting attractors can be revealed when a_0_ = 1.75; a_1_ = b = 3; a_3_ = 1; *γ* = 0.0109 and the control parameter a_2_ is varied in a tiny range. The bifurcation diagrams of Figure 12 revealed a window in which up to seven disconnected attractors (see Figure 13) coexist in the phase space depending solely on the system's initial state.  Table 2 provides the initial conditions for these coexisting attractors.

This work represents an enriching contribution to the understanding of the nonlinear dynamics of this type of oscillators [36]. However, this striking phenomenon of disconnected coexisting attractors is also reported in other nonlinear dynamic systems such as lazer system [37], chemical reaction [38], and the radio physical system [35]. A special case where infinitely many attractors coexist, also referred to as extreme multistability, is discussed in [26, 39]. The multiplicity of attractors represents an additional type of randomness [40] that is exploited in real applications such as chaos based secret communication, image encryption, and random signal generation as well. However, this type of behavior is not desirable in general, thus the need for control. Detailed analysis on this line is out of the scope of this paper. Also, we suggest the excellent work on control of multistability by [41] to interested readers.

## 4. Pspice Circuit Simulations

Our motivation in this section is to verify the theoretical/numerical results obtained previously by performing some Pspice based simulations of the circuit. Furthermore, it is important to evaluate the effects of simplifying assumptions (e.g., ideal diode model and ideal op. amplifiers) considered during the mathematical modeling process, on the behavior of a hardware prototype of the 5-D hyperjerk circuit in Pspice. Briefly recall that an interesting aspect of using Pspice is the possibility of setting initial capacitors' voltages and analyzing the corresponding influence on the dynamics of the complete electronic circuit. Thus, the presence of multiple coexisting attractors can be tracked in a straightforward manner.

First to report the reverse period doubling routes to chaos observed during the numerical analysis, the circuit of Figure 1 has been simulated with the following electronic components: R = 10k*Ω*, C = 10*η*F, *R*_*a*0_ = 5.71*kΩ*, *R*_*a*1_ = *R*_*b*_ = 3.33*kΩ*, *R*_*a*3_ = 10*kΩ*. By varying *R*_*a*2_, the complete routes to chaos are obtained and depicted in Figure 3-right. For *R*_*a*2_ = 15.5*kΩ* a period-1 limit cycle is obtained, for *R*_*a*2_ = 16.2*kΩ* a period-2 limit cycle is obtained, and chaotic attractors are obtained for *R*_*a*2_ = 17*kΩ* and *R*_*a*2_ = 18*kΩ*.

**Figure 14:**
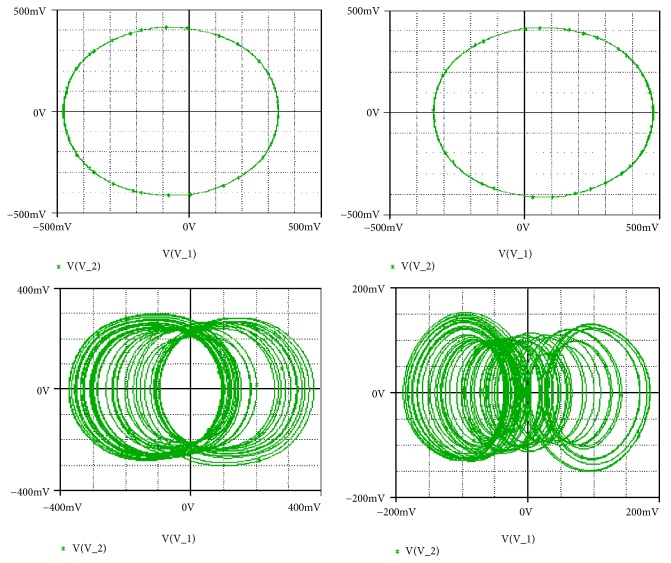
Pspice simulation results showing the coexistence of four different attractors for *Ra*2 = 17.91*kΩ* (a pair of period-1 limit cycles, and two symmetric chaotic attractors). Initial states are indicated in Table 1.

**Figure 15:**
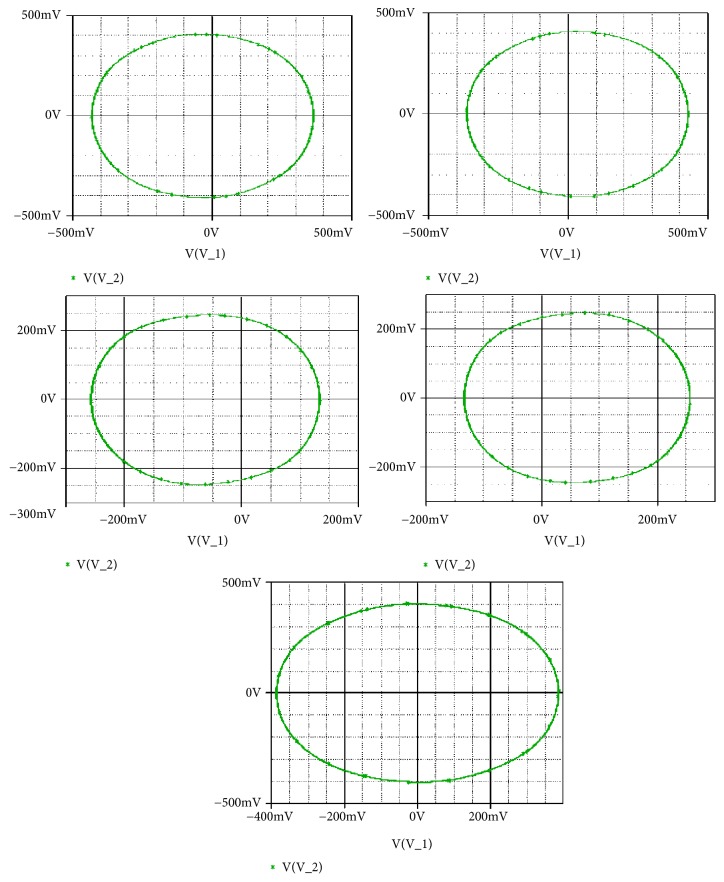
Pspice simulation results showing the coexistence of five different attractors for *Ra*2 = 14.65*kΩ* (a pair of chaotic attractors, a pair of period-1 limit cycle, and a symmetric chaotic attractor). Initial states are indicated in Table 1.

**Figure 16:**
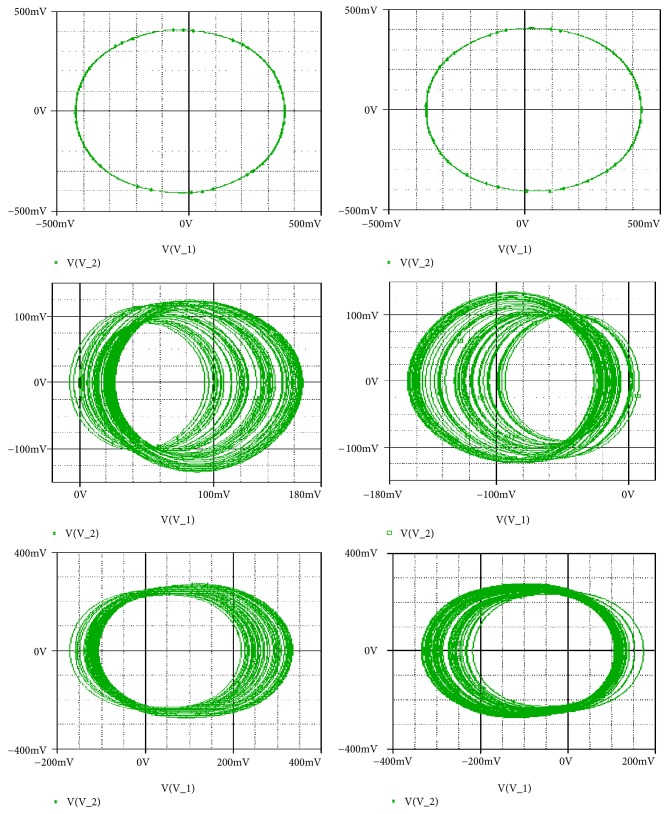
Pspice simulation results showing the coexistence of six different attractors for *Ra*2 = 16.5*kΩ* (two pairs of chaotic attractors and a pair of period-1 limit cycle). Initial states are indicated in Table 1.

Secondly, coexistence of multiple attractors can also be confirmed by Pspice based simulations with the following electronic circuit components: R = 50k*Ω*, C = 2*η*F, Ra_0_ = 28.57k*Ω*; R_a__1_ = 16.66k*Ω*; R_a__3_ = 50k*Ω*; R_b_ = 16.66k*Ω*. If *R*_*a*2_ is fixed to *R*_*a*2_ = 17.91*kΩ*, four disconnected chaotic and periodic attractors (see Figure 14) coexist depending solely on the system's initial states as indicated in Table 2. For *R*_*a*2_ = 15.6*kΩ*, five different period-1 limit cycles can be observed (see Figure 15). The corresponding initial states are indicated in Table 2. If *R*_*a*2_ is fixed to *R*_*a*2_ = 16.5*kΩ*, six disconnected chaotic and periodic attractors (see Figure 16) coexist depending solely on the system's initial states as indicated in Table 2. A very good similarity between numerical phase portraits and Pspice simulation results can be observed. However, slight discrepancies that may be attributed to the simplifying assumptions adopted during the modeling process can be noted between the bifurcations points in Pspice compared to the results from the theoretical analysis. It is important to stress that while Pspice software is based on actual circuit components, it still suffers from the discretization and its use can lead to wrong conclusions especially for hidden attractors (the same is true for MATLAB) [42].

## 5. Conclusion

A novel 5-D hyperjerk circuit with a very simple nonlinear part has been introduced in this work. The circuit is obtained by introducing additional feedback loops in the realization circuit of a jerk system previously reported by J. Kengne and collaborators. The modification yields the simplest 5-D hyperjerk system reported up to date. More interestingly, for some given sets of parameters, the system experiences a plethora of multiple coexisting attractors. For instance, up to seven disconnected attractors coexist in the system depending solely on the initial conditions. To the best of author's knowledge, such dynamics has not yet been reported in any hyperjerk system and thus deserves dissemination. Pspice based simulations were carried out to support the theoretical analysis. A detailed exploration of the parameter space (both experimentally and numerically) in view of revealing hyperchaotic behavior and hidden attractors in system (3) deserves further studies.

## Figures and Tables

**Table 1:** Comparative analysis of some dynamical systems by using the largest Lyapunov exponent (*λ*max).

***References***	***Dynamical system***	***Parameters***	***Maximum Lyapunov exponent*** *λ*_max_
***[16]***	${\dot{x}}_{1}=a(x_{2}-x_{1})+x_{4}+x_{5}$	*a* = 10	*λ*_max_ = 0.4195
	${\dot{x}}_{2}=cx_{1}-x_{1}x_{3}-x_{2}$	$b=\frac{8}{3}$	
	${\dot{x}}_{3}=x_{1}x_{2}-bx_{3}$	*c* = 28, *p* = 1.3	
	${\dot{x}}_{4}=-x_{1}x_{3}+px_{4}$	*q* = 2.5	
	${\dot{x}}_{5}=qx_{1}$		

***[17]***	${\dot{x}}_{1}=x_{2}$	*a* = 4	*λ*_max_ = 0.1589
	${\dot{x}}_{2}=ax_{3}$	*γ* = 0.06, *μ* = 0.75	
	${\dot{x}}_{3}=-\gamma x_{2}-\mu x_{3}+x_{1}-x_{1}\left|x_{1}\right|$		

***[21]***	${\dot{x}}_{1}=-x_{2}-x_{3}$	*a* = 0.25, *b* = 3	*λ*_max_ = 0.112
	${\dot{x}}_{2}=x_{1}+ax_{2}+x_{4}$	*c* = 0.5, *d* = 0.05	
	${\dot{x}}_{3}=b+x_{1}x_{3}$		
	${\dot{x}}_{4}=-cx_{3}+dx_{4}$		

***[23]***	${\dot{x}}_{1}=a(x_{2}-x_{1})+x_{4}$	*a* = 10, *r* = 28	*λ*_max_ = 0.39854
	${\dot{x}}_{2}=cx_{1}-x_{1}x_{3}-x_{2}$	$b=\frac{8}{3},\;\;d=1.3$	
	${\dot{x}}_{3}=x_{1}x_{2}-bx_{3}$		
	${\dot{x}}_{4}=-x_{1}x_{3}+dx_{4}$		

***This work***	${\dot{x}}_{1}=x_{2}$	*b*≔32	*λ*_max_ = 0.82
	${\dot{x}}_{2}=x_{3}$	*a*_0_ = 5, *a*_1_ = 7	
	${\dot{x}}_{3}=x_{4}$	*a*_2_ = 5, *a*_3_ = 4.50	
	${\dot{x}}_{4}=bx_{5}$	*a*_4_ = 5.4433 × 10^−4^	
	${\dot{x}}_{5}=-a_{0}x_{5}-a_{1}x_{3}-a_{2}x_{2}-a_{3}x_{1}-\gamma \sinh\left(x_{4}\right)$		

**Table 2:** Initial conditions for the abundant coexisting attractors.

***Figures***	***Type of coexistence***	***Dimensionless parameter***	***Corresponding electronics components***	***Numerical initial conditions*** (*x*_10_, *x*_20_, *x*_30_, *x*_40_, *x*_50_)	***Pspice initial conditions*** (*υ*_10_, *υ*_20_, *υ*_30_, *υ*_40_, *υ*_50_)
6	Four disconnected chaotic attractors	*b* = 29.18	*R*_*b*_ = 342.7*Ω*	(a) (1,0, 0,0, 0) (b) (3,0, 0,0, 0)	–

8 ***&*** 14	Four disconnected chaotic and periodic attractors	*a*_2_ = 2.458	*R*_*a*2_ = 17.91*kΩ*	(a) (±6,0, 0,0, 0) (b) (2,0, 0,0, 0) (c) (3,0, 0,0, 0)	(a-b) (±0.3,0, 0,0, 0) (c-d) (±1,0, 0,0, 0) (e) (0,0, 0.2,0, 0)

10	Five disconnected chaotic and periodic attractors	*a*_2_ = 2.71	*R*_*a*2_ = 14.65*kΩ*	(a) (±5,0, 0,0, 0) (b) (±0.6,0, 0,0, 0) (c) (5.6,0, 0,0, 0)	(a-b) (±0.2,0.1,0.1,0.2) (c-d) (±0.5,0.1,0.1,0.2) (c-d) (±0.5,0.1,0.1,0.2)

9 ***&*** 15	Five disconnected periodic attractors	*a*_2_ = 3	*R*_*a*2_ = 15.6*kΩ*	(a) (±5,0, 0,0, 0) (b) (±0.6,0, 0,0, 0) (c) (5.6,0, 0,0, 0)	(a-b) (±0.1,0, 0,0, 0) (c-d) (±0.2,0, 0,0, 0) (e-f) (±0.3,0, 0,0, 0)

11 ***&*** 16	Six disconnected chaotic and periodic attractors	*a*_2_ = 2.8	*R*_*a*2_ = 16.5*kΩ*	(a) (±0.45,0, 0,0, 0) (b) (±0.6,0, 0,0, 0) (c) (±0.35,0, 0,0, 0)	(a-b) (±0.1,0, 0,0, 0) (c-d) (±0.2,0, 0,0, 0) (e-f) (±0.3,0, 0,0, 0)

13	Seven disconnected chaotic and periodic attractors	*a*_2_ = 2.834	*R*_*a*2_ = 35.28*kΩ*	(a) (1,0, 0,0) (b) (±3,0, 0,0) (c) (±2,0, 0,0) (d) (4,0, 0,0)	–
